# Chemical reaction and thermal radiation impact on a nanofluid flow in a rotating channel with Hall current

**DOI:** 10.1038/s41598-021-99214-y

**Published:** 2021-10-05

**Authors:** Yu-Pei Lv, Naila Shaheen, Muhammad Ramzan, M. Mursaleen, Kottakkaran Sooppy Nisar, M. Y. Malik

**Affiliations:** 1 Department of Mathematics, Huzhou University, Huzhou, 313000 People’s Republic of China; 2 Department of Computer Science, Bahria University, Islamabad, 44000 Pakistan; 3 Department of Medical Research, China Medical University Hospital, China Medical University (Taiwan), Taichung, Taiwan; 4 Department of Mathematics, Aligarh Muslim University, Aligarh, 202002 India; 5 Department of Mathematics, College of Arts and Sciences, Prince Sattam Bin Abdulaziz University, Wadi Aldawaser, 11991 Saudi Arabia; 6 Department of Mathematics, College of Sciences, King Khalid University, Abha, 61413 Saudi Arabia

**Keywords:** Mechanical engineering, Software

## Abstract

The objective of the present exploration is to examine the nanoliquid flow amid two horizontal infinite plates. The lower plate is stretchable and permeable. The uniqueness of the flow model is assimilated with the Hall effect, variable thermal conductivity, thermal radiation, and irregular heat source/sink. Transmission of mass is enhanced with the impression of chemical reaction incorporated with activation energy. Appropriate similarity transformation is applied to transform the formulated problem into ordinary differential equations (ODEs). The numerical solution is obtained by employing MATLAB software function bvp4c. The dimensionless parameters are graphically illustrated and discussed for the involved profiles. An increasing behavior is exhibited by the temperature field on escalating the Brownian motion, thermophoresis parameter, variable thermal conductivity, and radiation parameter. For larger values of Schmidt number and chemical reaction parameter, the concentration profile deteriorates, while a reverse trend is seen for activation energy. The rate of heat transfer is strengthened at the lower wall on amplifying the Prandtl number. A comparative analysis of the present investigation with already published work is also added to substantiate the envisioned problem.

## Introduction

Fluid flow in a rotating channel is immensely acknowledged because of its numerous applications in designing turbines, the structure of rotating magnetic stars, MHD generators, movement of oil and gas through the reservoir is observed by petroleum engineers, and flow of blood in the pulmonary alveolar sheet. Rotational flow can be seen in tropical cyclones, whirlpools, and tornadoes. Bilal et al.^1^ inspected Viscoelastic fluid embedded with dust particles in a rotating channel. It is delineated here that fluid temperature upsurges on amplifying the radiation parameter. On a magneto hybrid nanoliquid flow, Khan et al.^2^ numerically explored the influence of heat generation/absorption and activation energy. It is perceived that on strengthening the Prandtl number and radiation parameter rate of heat transfer diminishes. On a nanoliquid flow, the aftermath of melting heat and radiative flux is addressed by Giri et al.^3^ amid two infinite horizontal plates. Here, it this exploration it is concluded that the temperature of fluid hikes on escalating the rotating factor, whereas, an opposite behavior is seen for rising values of melting factor. Feroz et al.^4^ explored the significance of the Hall and ion slip effect on single-wall carbon nanotubes and multi-wall carbon nanotubes in a rotating channel. It is reported that the velocity of nanoliquid upsurges on mounting the hall and ion slip parameter. Hall and slip effect on a time-dependent laminar flow is discussed by Khan et al.^5^ in a rotating channel. Substantial research on a rotating channel with several physical aspects is cited in^6–16^.

Variable heat source and sink play a vital role in the exclusion of heat from the rubble of nuclear fuel, cooling of metallic sheets, discard waste radioactive material, radial diffusers, and unpolished oil retrieval. On a laminar Micropolar fluid flow impact of a non-uniform heat source/sink is numerically analyzed by Singh et al.^17^ with variable thermal conductivity amongst an inclined channel. It is concluded here that the velocity and the temperature field reduce on augmenting the material parameter. Darcy Forchheimer flow incorporated with irregular heat source/sink is assessed by Upreti et al.^18^ on a 3D magnetohydrodynamic (MHD) flow of carbon nanotubes on an elongated sheet. It is comprehended here that the rate of heat transfer enhances elevating the concentration of nanoparticles. Srinivasulu and Bandari^19^ illustrated the outcome of irregular heat source/sink on Williamson nanoliquid flow amalgamated with non-linear thermal radiation on an inclined deforming surface. It is perceived an opposite behavior in the concentration field for Brownian and thermophoresis parameter. The impact of irregular heat source/sink, Joule dissipation is explored by Thumma and Mishra^20^ on a 3D Eyring-Powell nanofluid on a deformable surface. In this study, it is noticed that fluid velocity upsurges for the rising fluid parameter. Khan et al.^21^ studied the impact of Darcy Forchheimer on a micropolar nanofluid in a rotating flow between two parallel plates. Here, it is noted that fluid velocity diminishes on escalating the porosity parameter. Recent endeavors with variable heat source and sink past a deformable surface are mentioned in^22–24^.

In a chemical reaction, reactants require minimum energy to prompt a reaction is known as activation energy. Fluid flow amalgamated with chemical reaction and activation energy has widespread applications which include the destruction of harvests due to freezing, manufacturing of paper, food processing, ceramics, drying, dehydration processes, oil, and water emulsions. Khan et al.^25^ analytically examined the Buongiorno model with viscous dissipation incorporated with chemical reaction and activation energy on a time-dependent second-grade nano liquid amid two infinite horizontal plates. It is reported that an increasing behavior is depicted by the temperature field on escalating the Brownian and thermophoresis parameter. Seyedi et al.^26^ formulated a model to numerically analyze the impact of a chemical reaction and linear thermal radiation on Eyring-Powell fluid on a squeezing deforming channel. It is stated here that fluid concentration enhances augmenting the fluid parameters. On a time-dependent, MHD third-grade nano liquid flow Chu et al.^27^ explored the influence of bio-convection, variable thermal conductivity coupled with activation energy past an elongated sheet. In this exploration, it is delineated that fluid concentration upsurges on amplifying the activation energy. The influence of the Buongiorno model on a Casson fluid is presented by Gireesha et al.^28^ past a stretchable sheet assimilated with non-linear thermal radiation and activation energy. It is observed that the growing values of non-linear radiative flux enhance the heat transfer rate. On a time-dependent, MHD Eyring Powell fluid flow the outcome of heat generation/absorption with chemical reaction is demonstrated by Ghadikolaei et al.^29^ past a stretchable channel. It is noticed that on augmenting the squeezing parameter the fluid temperature deteriorates. Recent studies on chemical reaction are cited in^15,30–35^.

Hall current is induced when the magnetic field is normal to the flow of the current. In the presence of a strong magnetic field, the phenomenon of Hall current is prominent. Due to this Ohm’s law is modified. The flow is changed to cross-flow thus making it three-dimensional. The impact of hall current has attracted great attention by researchers due to its usage as Hall sensors, thermal energy storage, Hall accelerators, MHD power generators, and turbines. In the field of medicine in medical tests such as cardiac magnetic resonance angiography, magnetic resonance imaging, etc. Saleem et al.^36^ formulated a 3D time-dependent upper convected Maxwell fluid model and investigated the outcome of the Hall effect, radiative flux, and heat generation /absorption past an elongated sheet. Here, it this exploration, it is reported that fluid temperature diminishes on augmenting the hall parameter. The impact of Hall current and ion slip parameter on a micropolar fluid past a vertical duct is discussed by Opanuga et al.^37^. It is perceived that primary and secondary velocity rises on enhancing the Hall effect parameter. On a radiative nanoliquid flow the outcome of hall current incorporated with porosity is deliberated by Mallick et al.^38^ in a wavy channel. It is concluded here that the temperature of nanoliquid augments for growing values of volume fraction of nanoparticle, however, a reverse trend is noticed for Hall current. Shah et al.^39^ analytically illustrated thermal relaxation properties in addition to Hall current on a couple of stress nano liquid over an exponentially deforming sheet. It is reported that on amplifying the Schmidt number the fluid concentration decays. Subsequently, exploration in this regard with different physical aspects can be seen in refs^16,40–42^.

The aforementioned studies illustrate that a great amount of research may be quoted that discusses the fluid flow in a rotating horizontal duct. The study of nanoliquid flow influenced by chemical reaction and activation energy in a rotating duct is still scarce and yet not discussed in the literature. The novelty of the problem is enriched by the addition of Hall current and linear radiation. The flow is analyzed under the impact of variable thermal conductivity and variable heat source/sink. The equations governing the mathematical problem are transformed into Ordinary differential equations (ODEs) by utilizing suitable similarity transformation. The mathematical model is deciphered through MATLAB software bvp4c. The outcome of numerous parameters is examined via tabular and graphical illustration. Innovation of the presented mathematical model is illustrated in Table 1 by associating it with the published studies.

**Table 1:** An inspection of literature for the innovation of the presented model.

Authors	Rotating channel	Hall effect	Buongiorno model	Temperature-dependent thermal conductivity	Thermal radiation	Non-uniform heat source/sink	Activation energy
Bilal et al.^1^	Yes	No	No	No	Yes	No	No
Khan et al.^2^	Yes	No	No	No	Yes	No	Yes
Seth et al.^8^	Yes	Yes	No	No	No	No	No
Mabood et al.^9^	Yes	Yes	Yes	No	No	No	No
Tlili et al.^43^	Yes	Yes	Yes	No	Yes	No	No
Present	Yes	Yes	Yes	Yes	Yes	Yes	Yes

## Mathematical problem formulation

**Figure 1:**
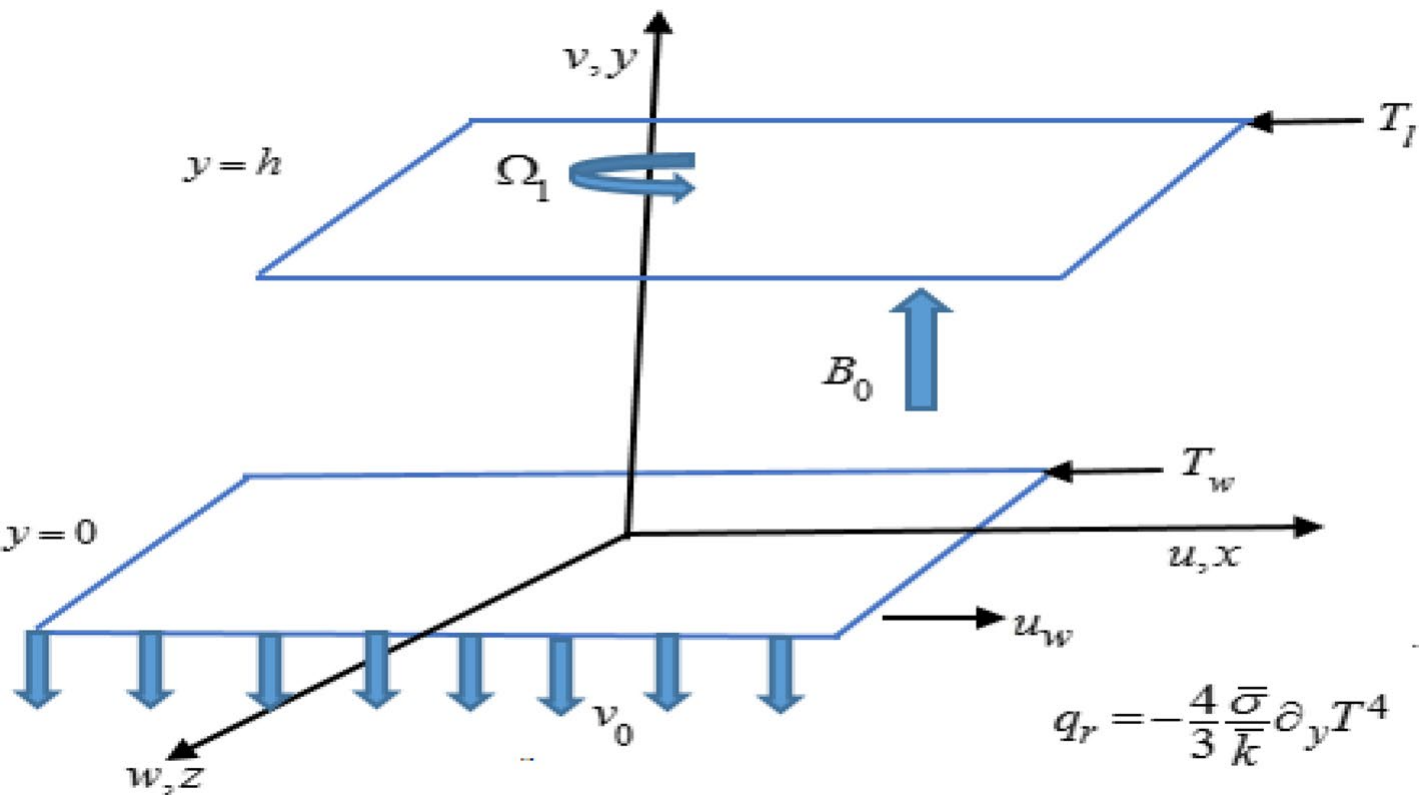
Schematic illustration of the flow.

An incompressible, laminar flow of a nanofluid in a rotating duct with an angular velocity $\Omega = \left( {0,\Omega_{1} ,0} \right)$ along the $y - {\text{axis}}$ is examined between two infinite horizontal plates with Hall current and thermal radiation. The nanofluid model describes the attributes of Brownian motion and thermophoresis. For the geometry of the problem, the Cartesian coordinate system is considered in such a manner that $x - {\text{axis}}$ is parallel to the plates, $y - {\text{axis}}$ is in the normal direction, whereas, $z - {\text{axis}}$ is transverse to the $xy - {\text{plane}}{.}$ A schematic illustration for the flow is portrayed in Fig. 1. The fluid is electrically conducting as a uniform magnetic field is applied along with the $y - {\text{axis}}{.}$ The lower plate at $y = 0$ is stretched linearly $u_{w} = cx$ in $x - {\text{direction,}}$ whereas, the upper plate is situated at $y = h.$ Fluid is sucked by the lower plate with velocity $v = - \nu_{0}$($v_{0} > 0$ corresponds to suction and $v_{0} < \, 0$ for injection). Transmission of heat and mass is boosted with the impression of temperature-dependent thermal conductivity, variable heat source/sink combined with chemical reaction, and activation energy.

The equations governing the flow of nanoliquid are^2,3,44^:

1 $$ \partial_{x} \tilde{u} + \partial_{y} \tilde{v} = 0, $$

2 $$ \tilde{u}\partial_{x} \tilde{u} + \tilde{v}\partial_{y} \tilde{u} + 2\Omega_{1} \tilde{w} = - \frac{1}{\rho }\partial_{x} p + \nu \left( {\partial_{xx} \tilde{u} + \partial_{yy} \tilde{u}} \right) + \frac{{\sigma_{1} B_{0}^{2} }}{{\rho \left( {1 + m^{2} } \right)}}\left( {\tilde{u} - m\tilde{w}} \right), $$

3 $$ \tilde{u}\partial_{x} \tilde{v} + \tilde{v}\partial_{y} \tilde{v} = - \frac{1}{\rho }\partial_{y} p + \nu \left( {\partial_{xx} \tilde{v} + \partial_{yy} \tilde{v}} \right), $$

4 $$ \tilde{u}\partial_{x} \tilde{w} + \tilde{v}\partial_{y} \tilde{w} - 2\Omega_{1} \tilde{u} = \nu \left( {\partial_{xx} \tilde{w} + \partial_{yy} \tilde{w}} \right) - \frac{{\sigma_{1} B_{0}^{2} }}{{\rho \left( {1 + m^{2} } \right)}}\left( {m\tilde{u} + \tilde{w}} \right), $$

5 $$ \begin{gathered} \tilde{u}\partial_{x} \tilde{T} + \tilde{v}\partial_{y} \tilde{T} = \frac{1}{{\rho c_{p} }}\partial_{y} \left[ {k\left( T \right)\partial_{y} \tilde{T}} \right] + \tau \left[ {D_{B} \partial_{y} \tilde{C}\partial_{y} \tilde{T} + \frac{{D_{T} }}{{\tilde{T}_{l} }}\left( {\partial_{y} \tilde{T}} \right)^{2} } \right] - \frac{1}{{\rho c_{p} }}\partial_{y} q_{r} \hfill \\ + \frac{{k\left( T \right)u_{w} }}{{x\nu \rho c_{p} }}\left[ {D\left( {\tilde{T}_{w} - \tilde{T}_{l} } \right)f^{\prime} + H\left( {\tilde{T} - \tilde{T}_{l} } \right)} \right], \hfill \\ \end{gathered} $$

6 $$ \tilde{u}\partial_{x} \tilde{C} + \tilde{v}\partial_{y} \tilde{C} = D_{B} \left[ {\partial_{xx} \tilde{C} + \partial_{yy} \tilde{C}} \right] + \frac{{D_{T} }}{{\tilde{T}_{l} }}\left( {\partial_{xx} \tilde{T} + \partial_{yy} \tilde{T}} \right) - k_{r}^{2} \left( {\tilde{C} - \tilde{C}_{l} } \right)\left( {\frac{{\tilde{T}}}{{\tilde{T}_{l} }}} \right)^{n} \exp \left( {\frac{{ - E_{a} }}{{k\tilde{T}}}} \right) $$

with boundary conditions^4,43–45^

7 $$ \begin{gathered} \left. {\tilde{u}} \right|_{y = 0} = u_{w} = cx, \, \left. {\tilde{v}} \right|_{y = 0} = - v_{0} , \, \left. {\tilde{w}} \right|_{y = 0} = 0, \, \left. {\tilde{T}} \right|_{y = 0} = \tilde{T}_{w} , \, \left. {\tilde{C}} \right|_{y = 0} = \tilde{C}_{w} \hfill \\ \left. {\tilde{u}} \right|_{y = h} = 0,\left. {\tilde{v}} \right|_{y = h} = 0,\left. {\tilde{w}} \right|_{y = h} = 0,\left. {\tilde{T}} \right|_{y = h} = \tilde{T}_{l} ,\left. {\tilde{C}} \right|_{y = h} = \tilde{C}_{l} . \hfill \\ \end{gathered} $$

The mathematical form of radiative heat flux^44^ is as follow:

8 $$ q_{r} = - \frac{4}{3}\frac{{\overline{\sigma }}}{{\overline{k}}}\partial_{y} T^{4} ,\,{\text{where}}\,T^{4} = 4T_{l}^{3} T - 3T_{l}^{4} $$

In Eq. (5) thermal conductivity of the fluid varies with time^46,47^ and is stated as:

9 $$ k = k_{0} \left( {1 + d\left( {\frac{{T - T_{l} }}{{T_{w} - T_{l} }}} \right)} \right) $$

Utilizing Eqs. (8) and (9) in (5), we get

10 $$ \begin{gathered} \tilde{u}\partial_{x} \tilde{T} + \tilde{v}\partial_{y} \tilde{T} = \frac{1}{{\rho c_{p} }}\partial_{y} \left[ {k_{0} \left( {1 + d\theta } \right)\partial_{y} \tilde{T}} \right] + \tau \left[ {D_{B} \partial_{y} \tilde{C}\partial_{y} \tilde{T} + \frac{{D_{T} }}{{\tilde{T}_{l} }}\left( {\partial_{y} \tilde{T}} \right)^{2} } \right] + \frac{16}{3}\frac{{\overline{\sigma }}}{{\overline{k}}}\frac{{T_{l}^{3} }}{{\rho c_{p} }}\partial_{yy} T \hfill \\ + \frac{{k_{0} \left( {1 + d\theta } \right)u_{w} }}{{x\nu \rho c_{p} }}\left[ {D\left( {\tilde{T}_{w} - \tilde{T}_{l} } \right)f^{\prime} + H\left( {\tilde{T} - \tilde{T}_{l} } \right)} \right], \hfill \\ \end{gathered} $$

Using appropriate transformation^2,3,44^

11 $$ \tilde{u} = cxf^{\prime}\left( \zeta \right), \, \tilde{v} = - chf\left( \zeta \right), \, \tilde{w} = cxj\left( \zeta \right), \, \theta \left( \zeta \right) = \frac{{\tilde{T} - \tilde{T}_{l} }}{{\tilde{T}_{w} - \tilde{T}_{l} }}, \, \phi \left( \zeta \right) = \frac{{\tilde{C} - \tilde{C}_{l} }}{{\tilde{C}_{w} - \tilde{C}_{l} }}, \, \zeta = \frac{y}{h}. $$

By utilizing the above transformation Eq. (1) is trivially equated. However, Eqs. (2–4, 6, 7, and 10) take the form:

12 $$ \frac{{d^{4} f}}{{d\zeta^{4} }} = R_{e} \left( {\frac{df}{{d\zeta }}\frac{{d^{2} f}}{{d\zeta^{2} }} - f\frac{{d^{3} f}}{{d\zeta^{3} }}} \right) + 2\alpha_{1} \frac{dj}{{d\zeta }} - \frac{{Ha^{2} }}{{\left( {1 + m^{2} } \right)}}\left( {\frac{{d^{2} f}}{{d\zeta^{2} }} - m\frac{dj}{{d\zeta }}} \right), $$

13 $$ \frac{{d^{2} j}}{{d\zeta^{2} }} = R_{e} \left( {f\frac{dj}{{d\zeta }} - \frac{df}{{d\zeta }}j} \right) - 2\alpha_{1} \frac{df}{{d\zeta }} + \frac{{Ha^{2} }}{{\left( {1 + m^{2} } \right)}}\left( {j + m\frac{df}{{d\zeta }}} \right), $$

14 $$ \begin{gathered} \left( {\left( {\left( {1 + d\theta } \right) + \frac{4}{3}Rd} \right)R_{e} } \right)\frac{{d^{2} \theta }}{{d\zeta^{2} }} = - \Pr \left( {f\frac{d\theta }{{d\zeta }} + R_{e} \left( {Nb\frac{d\theta }{{d\zeta }}\frac{d\phi }{{d\zeta }} + Nt\left( {\frac{d\theta }{{d\zeta }}} \right)^{2} } \right)} \right) \hfill \\ - \left( {1 + d\theta } \right)\left( {D\frac{df}{{d\zeta }} + H\frac{d\theta }{{d\zeta }}} \right) - R_{e} d\left( {\frac{d\theta }{{d\zeta }}} \right)^{2} , \hfill \\ \end{gathered} $$

15 $$ \frac{{d^{2} \phi }}{{d\zeta^{2} }} = - \frac{Nt}{{Nb}}\frac{{d^{2} \theta }}{{d\zeta^{2} }} + Sc\left( {\delta \phi \left( {1 + \alpha \theta } \right)^{n} \exp \left( {\frac{ - E}{{\left( {1 + \alpha \theta } \right)}}} \right) - f.R_{e} \frac{d\phi }{{d\zeta }}} \right). $$

boundary conditions take the form.

$$ \begin{gathered} {\text{Lower}}\;{\text{plate}}:\frac{df}{{d\zeta }}\left( 0 \right) = 1, \, f\left( 0 \right) = K, \, j\left( 0 \right) = 0, \, \theta \left( 0 \right) = 1, \, \phi \left( 0 \right) = 1, \hfill \\ {\text{Upper}}\;{\text{plate}}:\frac{df}{{d\zeta }}\left( 1 \right) = 0, \, f\left( 1 \right) = 0, \, j\left( 1 \right) = 0, \, \theta \left( 1 \right) = 0, \, \phi \left( 1 \right) = 0. \hfill \\ \end{gathered} $$

The mathematical forms of shear stress at the walls, local Nusselt and Sherwood number are specified as:

17 $$ C_{f,lower} = \frac{\mu }{{\rho u_{w}^{2} }}\left. {\partial_{y} u} \right|_{y = 0} $$

18 $$ C_{f,upper} = \frac{\mu }{{\rho u_{w}^{2} }}\left. {\partial_{y} u} \right|_{y = h} $$

19 $$ Nu_{lower} = \frac{{hQ_{w} }}{{k_{0} \left( {T_{w} - T_{l} } \right)}},\quad Q_{w} = \left. { - k(T)\partial_{y} T + q_{r} } \right|_{y = 0} $$

20 $$ Nu_{upper} = \frac{{hQ_{w} }}{{k_{0} \left( {T_{w} - T_{l} } \right)}},\quad Q_{w} = \left. { - k(T)\partial_{y} T + q_{r} } \right|_{y = h} $$

21 $$S{h_{lower}} = \frac{{h{Q_m}}}{{{D_B}\left( {{C_w} - {C_l}} \right)}},\,{Q_m} = {\left. { - {D_B}{\partial _y}C} \right|_{y = 0}}$$

22 $$S{h_{upper}} = \frac{{h{Q_m}}}{{{D_B}\left( {{C_w} - {C_l}} \right)}},\;{\mkern 1mu} {Q_m} = {\left. { - {D_B}{\partial _y}C} \right|_{y = h}}$$

By utilizing Eq. (11), Eq. (17–22) are transmuted as:

23 $${\left( {{{{\mathop{\rm Re}\nolimits} }_h}{C_f}} \right)_{{f_{lower}}}} = {\left. {\frac{{{d^2}f}}{{d{\zeta ^2}}}} \right|_{\zeta  = 0}},{\mkern 1mu} \,{\left( {{{{\mathop{\rm Re}\nolimits} }_h}{C_f}} \right)_{upper}} = {\left. {\frac{{{d^2}f}}{{d{\zeta ^2}}}} \right|_{\zeta  = 1}}$$

24 $$ \left( {Nu} \right)_{lower} = - \left( {1 + \left( \frac{4}{3} \right).\frac{Rd}{{1 + d\theta }}} \right)\left. {\frac{d\theta }{{d\zeta }}} \right|_{\zeta = 0} , \, \quad \left( {Nu} \right)_{upper} = - \left( {1 + \left( \frac{4}{3} \right).\frac{Rd}{{1 + d\theta }}} \right)\left. {\frac{d\theta }{{d\zeta }}} \right|_{\zeta = 1} $$

25 $$ (Sh)_{lower} = - \left. {\frac{d\phi }{{d\zeta }}} \right|_{\zeta = 0} , \, \quad (Sh)_{upper} = - \left. {\frac{d\phi}{{d\zeta }}} \right|_{\zeta = 1} $$

## Numerical procedure

The coupled nonlinear ODEs are computed numerically by employing the bvp4c function in MATLAB. Mentioned numerical code is used. Step size $h = 0.01$ is considered with the tolerance $10^{ - 6} ,$ respectively.

26 $$\begin{gathered} f = Y_{1} ,f^{\prime} = Y_{2} ,f = Y_{3} ,f^{\prime\prime\prime} = Y_{4} ,f^{iv} = Y_{4}^{\prime } = YY_{1} ,j = Y_{5} ,j^{\prime} = Y_{6} ,j = Y_{6} ^{\prime} = YY_{2} , \hfill \\ YY_{1} = \left[ {R_{e} \left( {Y_{2} \cdot Y_{3} - Y_{1} \cdot Y_{4} } \right) + 2\alpha_{1} \cdot Y_{6} - \left( {\frac{{Ha^{2} }}{{1 + m^{2} }}} \right)\left( {Y_{3} - m \cdot Y_{6} } \right)} \right], \hfill \\ YY_{2} = \left[ {R_{e} \left( {Y_{1} \cdot Y_{6} - Y_{2} \cdot Y_{5} } \right) - 2 \cdot \alpha_{1} \cdot Y_{2} + \left( {\frac{{Ha^{2} }}{{1 + m^{2} }}} \right)\left( {Y_{5} + m \cdot Y_{2} } \right)} \right], \hfill \\ \theta = Y_{7} ,\theta ^{\prime} = Y_{8} ,\theta = Y_{8}^{\prime } = YY_{3} ,\phi = Y_{9} ,\phi ^{\prime} = Y_{10} ,\phi = Y_{10}^{\prime } = YY_{4} , \hfill \\ YY_{3} = \frac{1}{{\left( {\left( {\left( {1 + d \cdot Y_{7} } \right) + \left( \frac{4}{3} \right)Rd} \right)R_{e} } \right)}}\left[ \begin{gathered} - \Pr \left( {Y_{1} \cdot Y_{8} + R_{e} \left( {N_{b} \cdot Y_{8} \cdot Y_{10} + N_{t} \cdot Y_{8} \cdot Y_{8} } \right)} \right) - \left( {1 + d \cdot Y_{7} } \right)\left( {D \cdot Y_{2} + H \cdot Y_{7} } \right) \hfill \\ - R_{e} \cdot d \cdot Y_{8} \cdot Y_{8} \hfill \\ \end{gathered} \right], \hfill \\ YY_{4} = \left[ { - \left( {\frac{{N_{t} }}{{N_{b} }}} \right) \cdot YY_{3} + \delta \cdot S_{c} \cdot Y_{9} \left( {1 + \cdot Y_{7} } \right)^{n} \exp \left( {\frac{ - E}{{1 + \delta \cdot Y_{7} }}} \right) - S_{c} \cdot R_{e} \cdot Y_{1} \cdot Y_{10} } \right] \hfill \\ {\text{and}}\,{\text{the}}\,{\text{boundary}}\,{\text{conditions}}\,{\text{are}} \hfill \\ {\text{At}}\,{\text{lower}}\,{\text{plate}}\quad Y_{1} (0) = K,Y_{2} (0) = 1,Y_{5} (0) = 0,Y_{7} (0) = 1,Y_{9} (0) = 1. \hfill \\ {\text{At}}\,{\text{upper}}\,{\text{plate }}\quad Y_{1} (1) = 0,Y_{2} (1) = 0,Y_{5} (1) = 0,Y_{7} (1) = 0,Y_{9} (1) = 0. \hfill \\ \end{gathered} $$

## Graphical results and discussion

**Figure 2:**
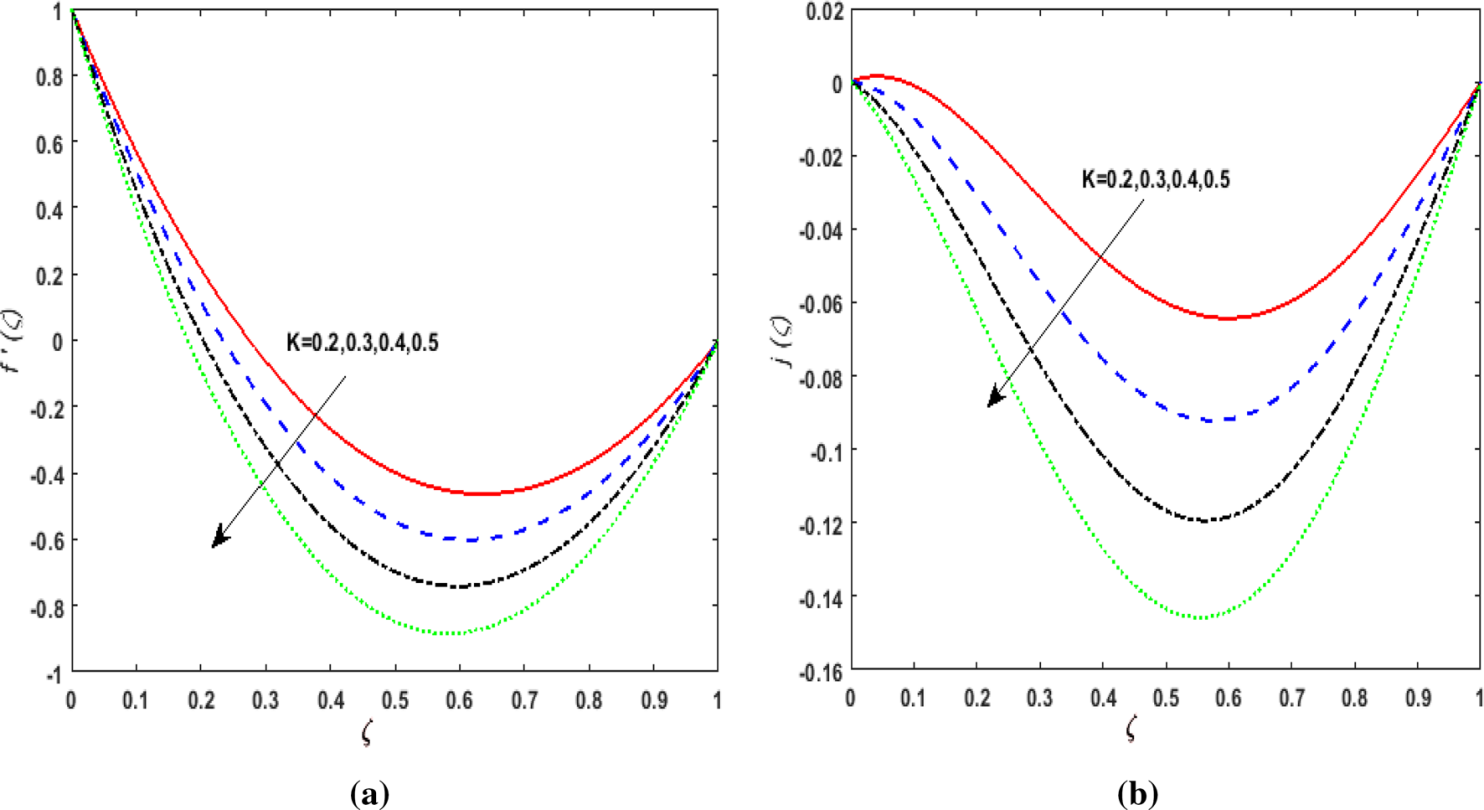
(**a**) Effect of suction parameter $K$ on velocity $f^{\prime}\left( \zeta \right)$. (**b**) Effect of suction parameter $K$ on velocity $j\left( \zeta \right)$.

**Figure 3:**
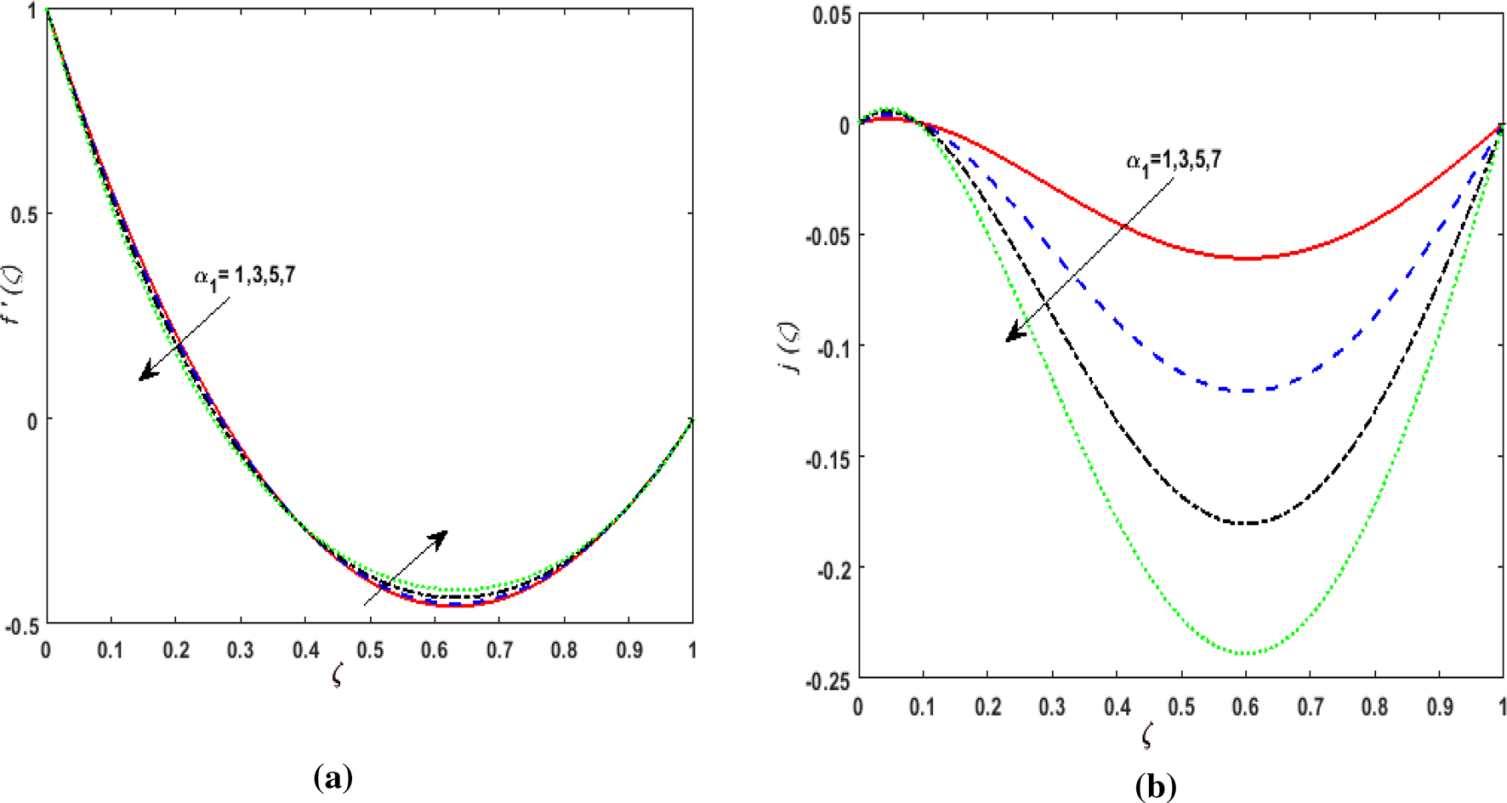
(**a**) Effect of rotation parameter $\alpha_{1}$ on velocity $f^{\prime}\left( \zeta \right)$. (**b**) Effect of rotation parameter $\alpha_{1}$ on velocity $j\left( \zeta \right)$.

**Figure 4:**
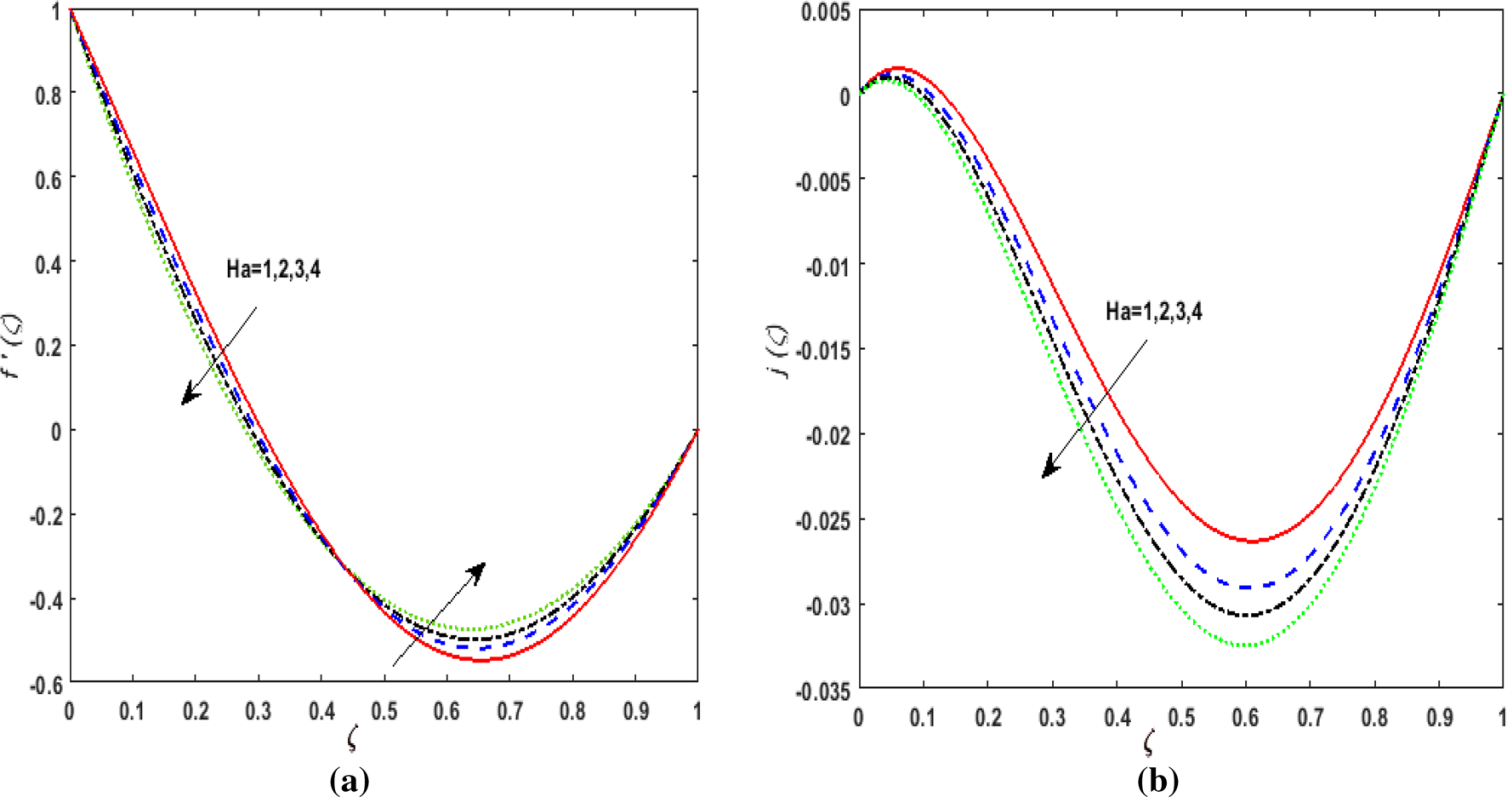
(**a**) Effect of magnetic parameter $Ha$ on velocity $f^{\prime}\left( \zeta \right)$. (**b**) Effect of magnetic parameter $Ha$ on velocity $j\left( \zeta \right)$.

**Figure 5:**
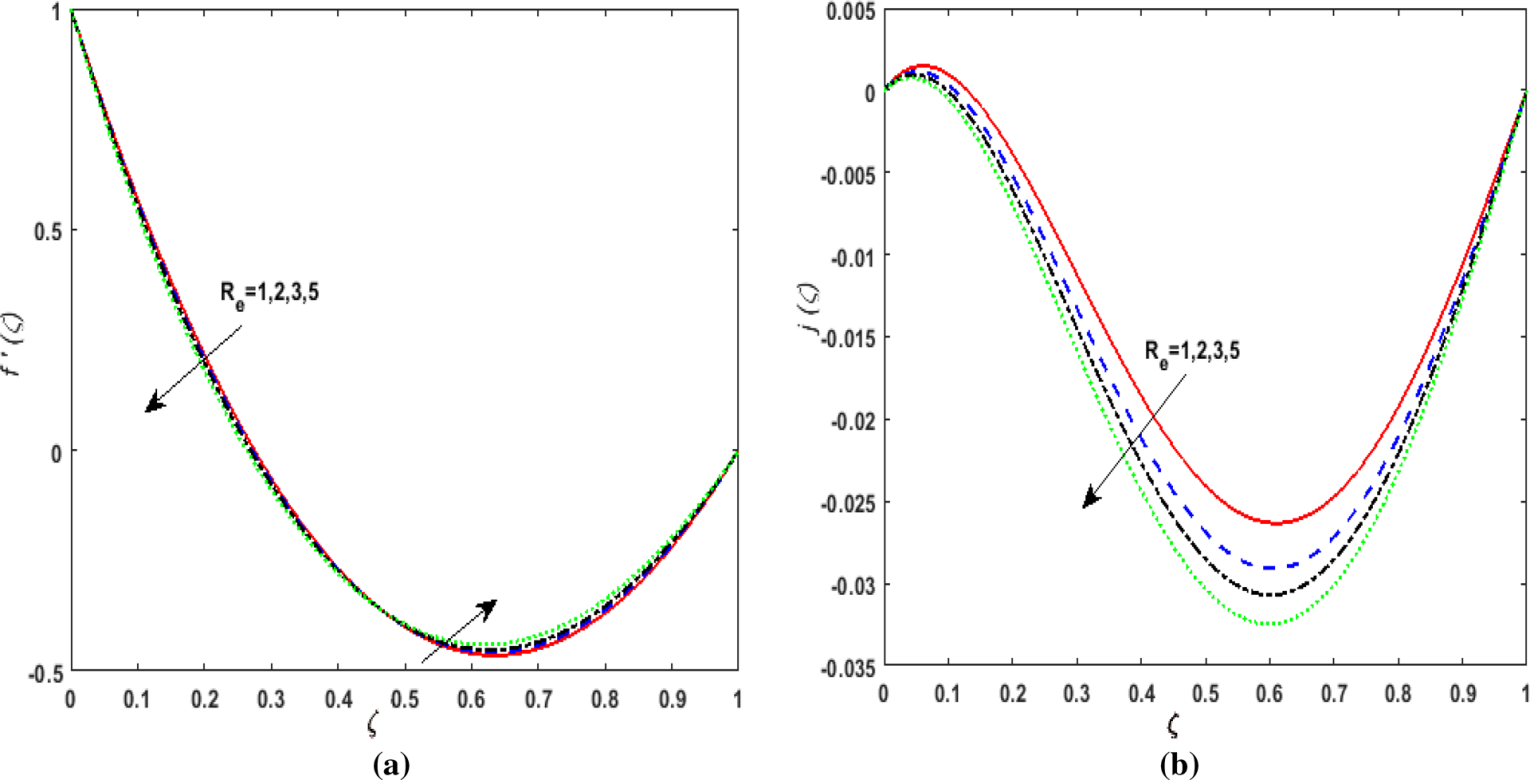
(**a**) Effect of Reynold number $R_{e}$ on velocity $f^{\prime}\left( \zeta \right)$. (**b**) Effect of Reynold number $R_{e}$ on velocity.

**Figure 6:**
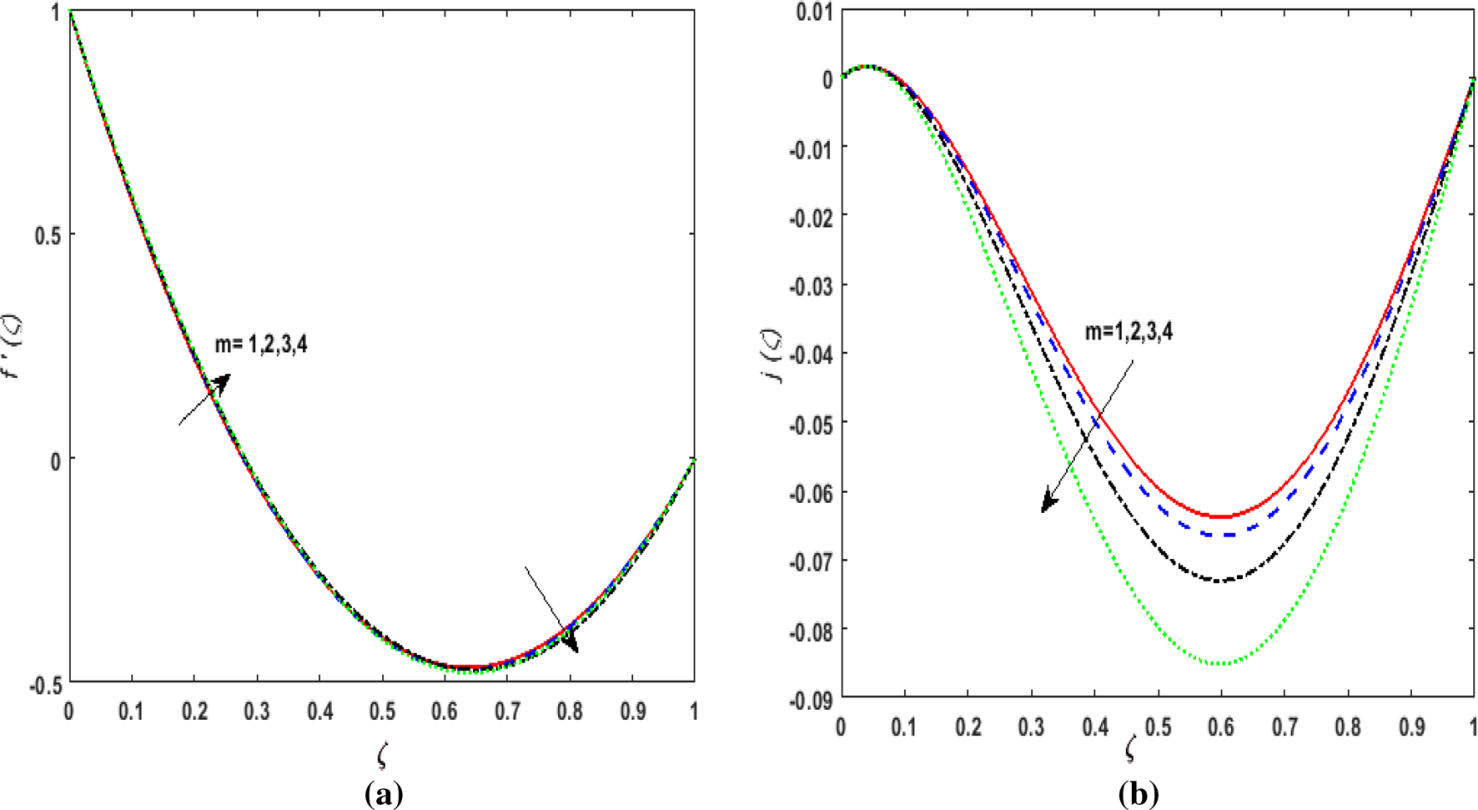
(**a**) Effect of hall parameter $m$ on velocity $f^{\prime}\left( \zeta \right)$. (**b**) Effect of hall parameter $m$ on velocity $j\left( \zeta \right)$.

The behavior of velocities $f^{\prime}\left( \zeta \right),j\left( \zeta \right)$, temperature $\theta \left( \zeta \right)$, and concentration $\phi \left( \zeta \right)$ is exhibited graphically for the dimensionless parameters appearing in the highly nonlinear mathematical problem in Eqs. (12)–(15). This problem is elucidated numerically by utilizing bvp4c, an implemented function in MATLAB. Consequently, additional pressure is developed in the fluid. The impact of varying the Suction parameter $K$ on both velocities are addressed in Fig. 2a and b. As nanoliquid is sucked by a lower plate which results in the ejection of a huge quantity of fluid in the vicinity of the lower plate. Thus, an impression of augmenting $K$ is witnessed as diminishing $f^{\prime}\left( \zeta \right)$ and $j\left( \zeta \right)$. The impact of the rotation parameter $\alpha_{1}$ on $f^{\prime}\left( \zeta \right)$ and $j\left( \zeta \right)$ is demonstrated in Fig. 3a and b. In a rotating channel, the motion of the fluid is opposed due to the Coriolis force which acts orthogonally to the velocity field and the rotational axis. Accordingly, a two-folded impression is noticed for $f^{\prime}\left( \zeta \right)$. On amplifying $\alpha_{1}$ the velocity $f^{\prime}\left( \zeta \right)$ decreases in the region close to the lower plate while a reverse trend is witnessed in the upper part of the channel. It is perceived that for growing values of $\alpha_{1}$ the velocity $j\left( \zeta \right)$ deteriorates. Figure 4a and b reflects the uplift of the magnetic parameter $Ha$ on $f^{\prime}\left( \zeta \right)$ and $j\left( \zeta \right)$. For mounting values of $Ha$, Lorentz force is strengthened which opposes the motion of the fluid. In Fig. 4a, initially, a downfall is noticed in $f^{\prime}\left( \zeta \right)$ before the mid-point of the channel nevertheless an augmenting nature is exhibited in the upper half of the channel. It is seen that on varying $Ha$ the velocity $j\left( \zeta \right)$ deteriorates. The impression of Reynold's number $R_{e}$ is elucidated in Fig. 5a and b. Since $R_{e}$ is the quotient of inertial forces to viscous forces. Therefore, on escalating $R_{e}$ inertial forces upsurges, whereas, viscous forces deteriorate. A two-folded influence is noticed for $f^{\prime}\left( \zeta \right)$, however, velocity $j\left( \zeta \right)$ deteriorates. The behavior of Hall current parameter $m$ on $f^{\prime}\left( \zeta \right)$ and $j\left( \zeta \right)$ is shown in Fig. 6a and b. By amplifying $m$ effective conductivity $\frac{{\sigma_{1} }}{{1 + m^{2} }}$ decreases. As a result effect of magnetic damping force is reduced. Thus in Fig. 6a, initially an upsurge is perceived in $f^{\prime}\left( \zeta \right)$ before the mid-point of the channel nevertheless a decreasing nature is exhibited in the lower half of the channel. Due to increment in $m$ fluid velocity $j\left( \zeta \right)$ declines.

**Figure 7:**
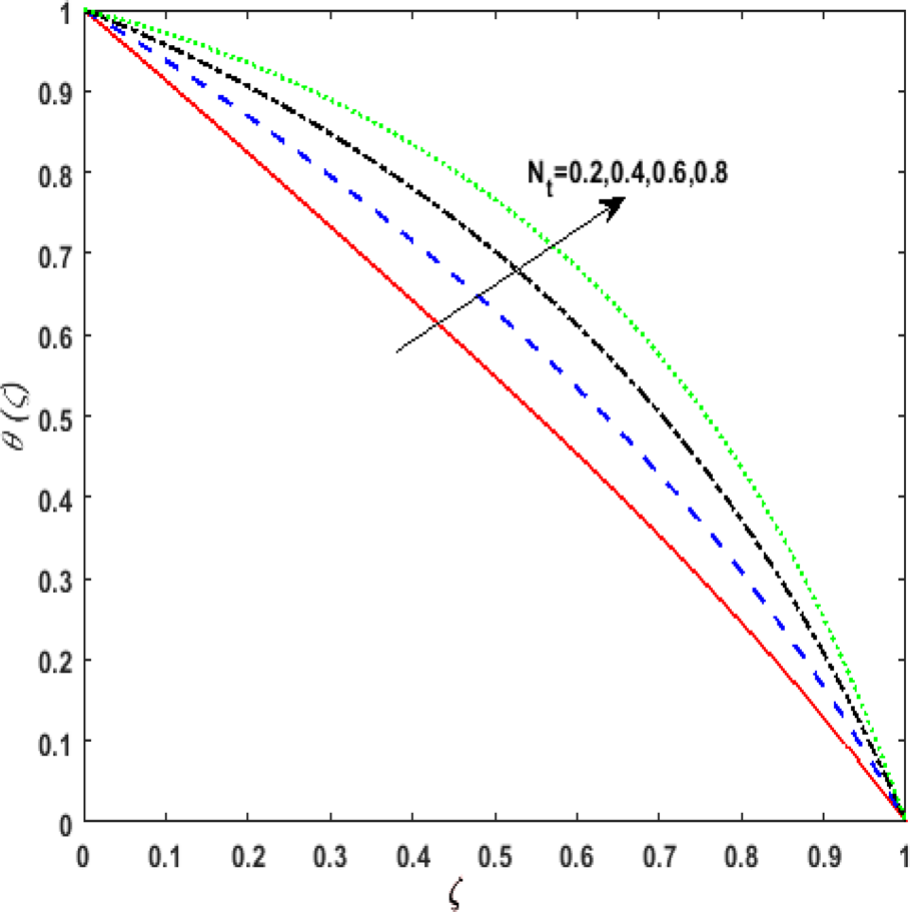
Effect of thermophoresis parameter $N_{t}$ on temperature $\theta \left( \zeta \right)$.

**Figure 8:**
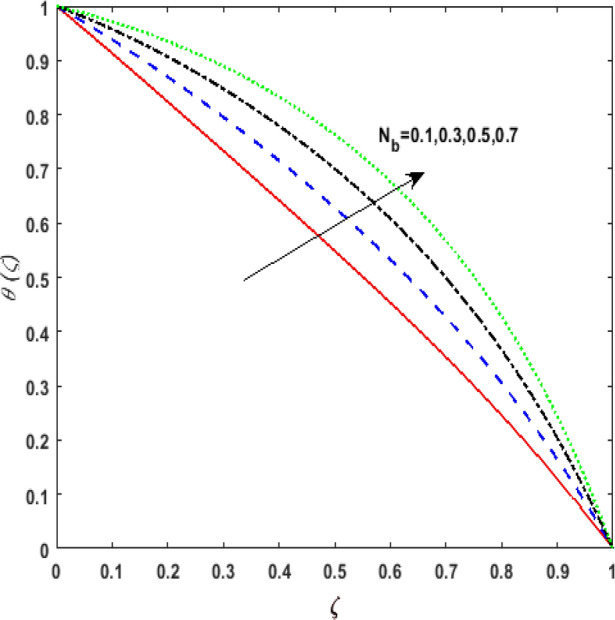
Effect of Brownian diffusion parameter $N_{b}$ on temperature $\theta \left( \zeta \right)$.

**Figure 9:**
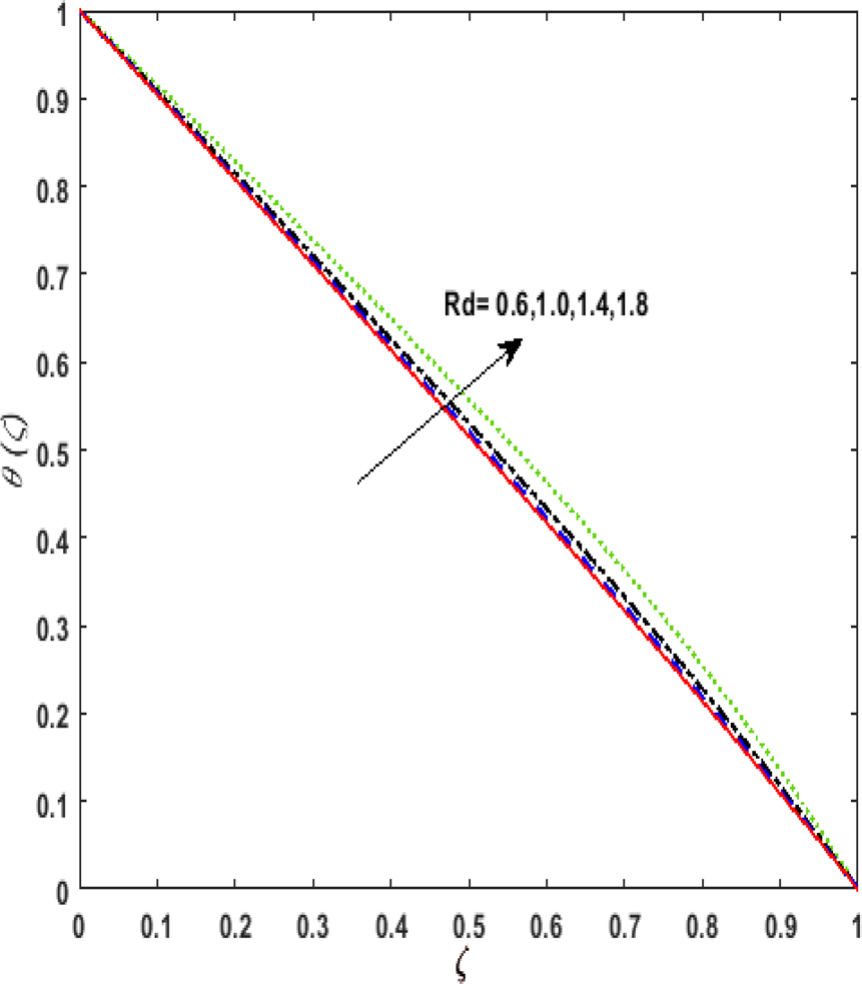
Effect of Radiation parameter $Rd$ on temperature $\theta \left( \zeta \right)$.

**Figure 10:**
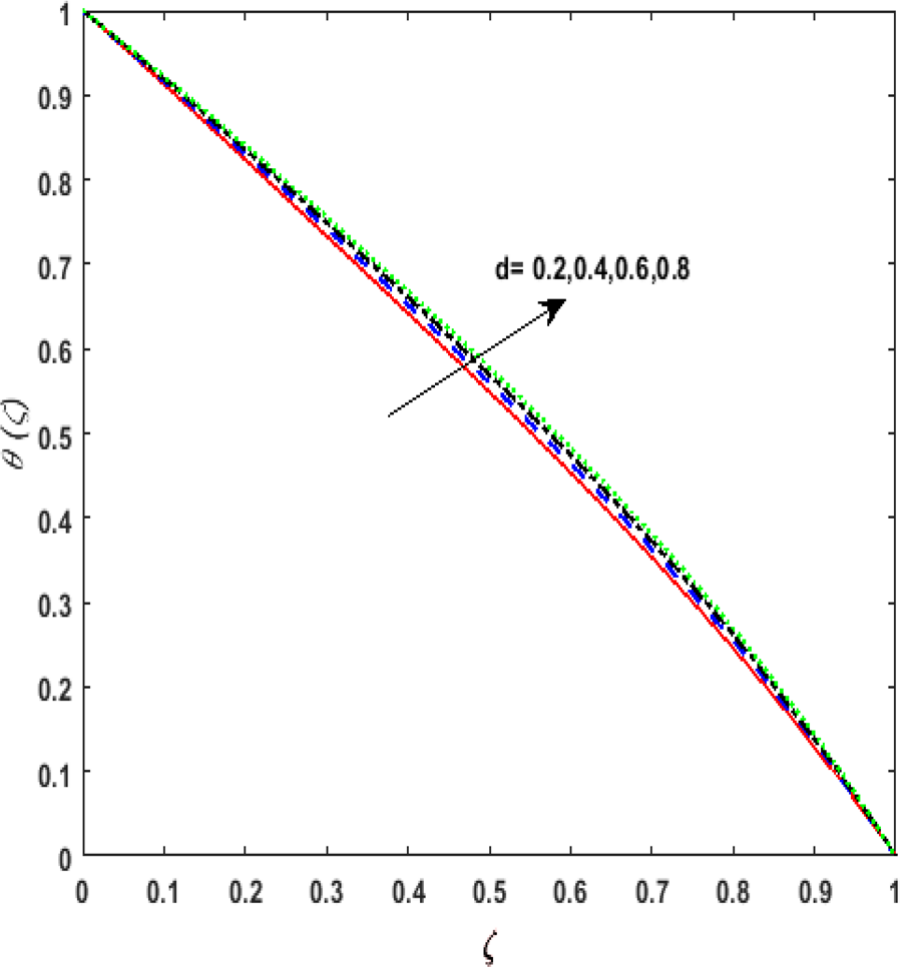
Effect of thermal conductivity parameter $d$ on temperature $\theta \left( \zeta \right)$.

**Figure 11:**
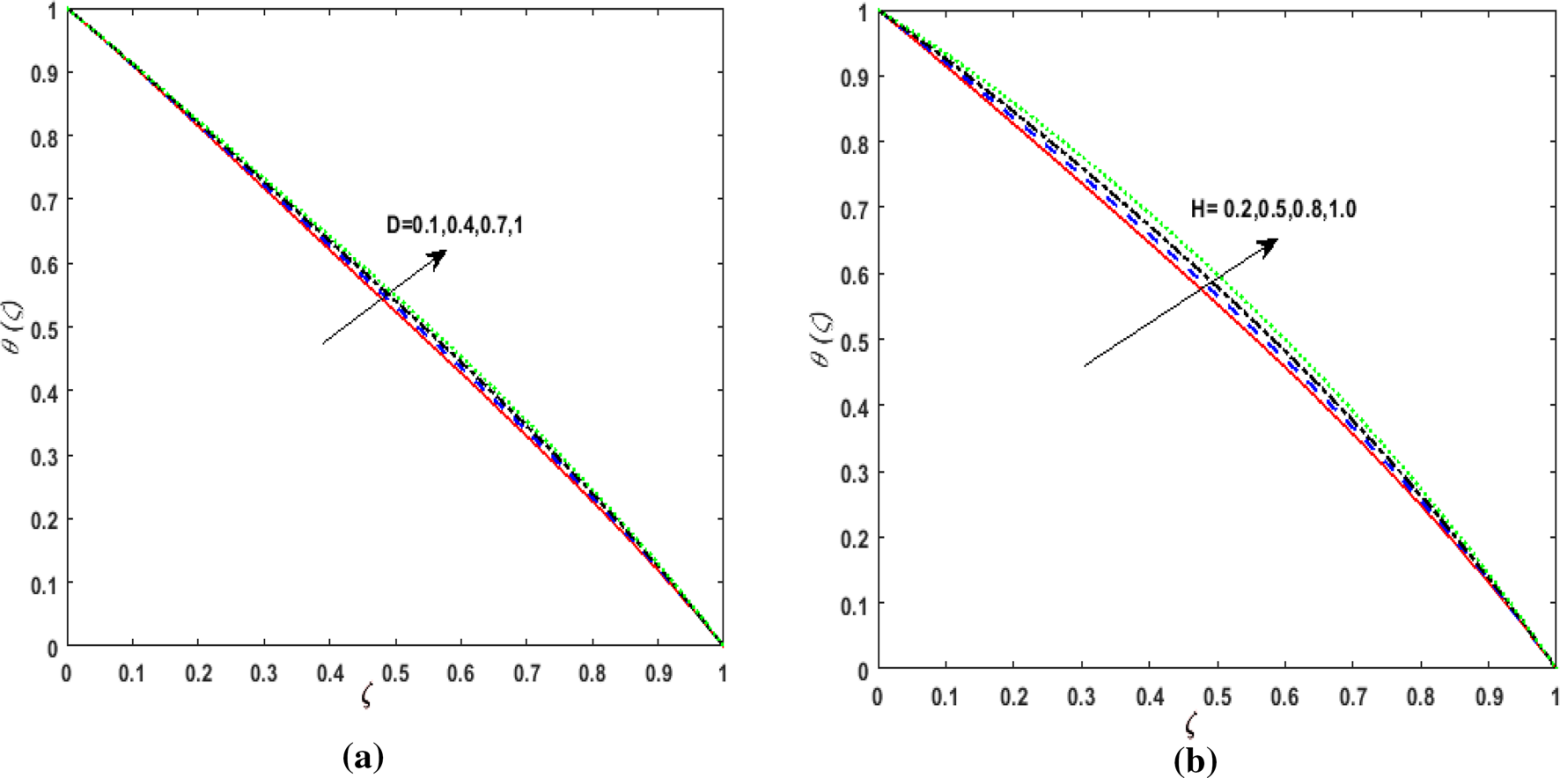
(**a**) Effect of temperature dependent source parameter $D > 0$ on temperature $\theta \left( \zeta \right)$. (**b**) Effect of space dependent source parameter $H > 0$ on temperature $\theta \left( \zeta \right)$.

**Figure 12:**
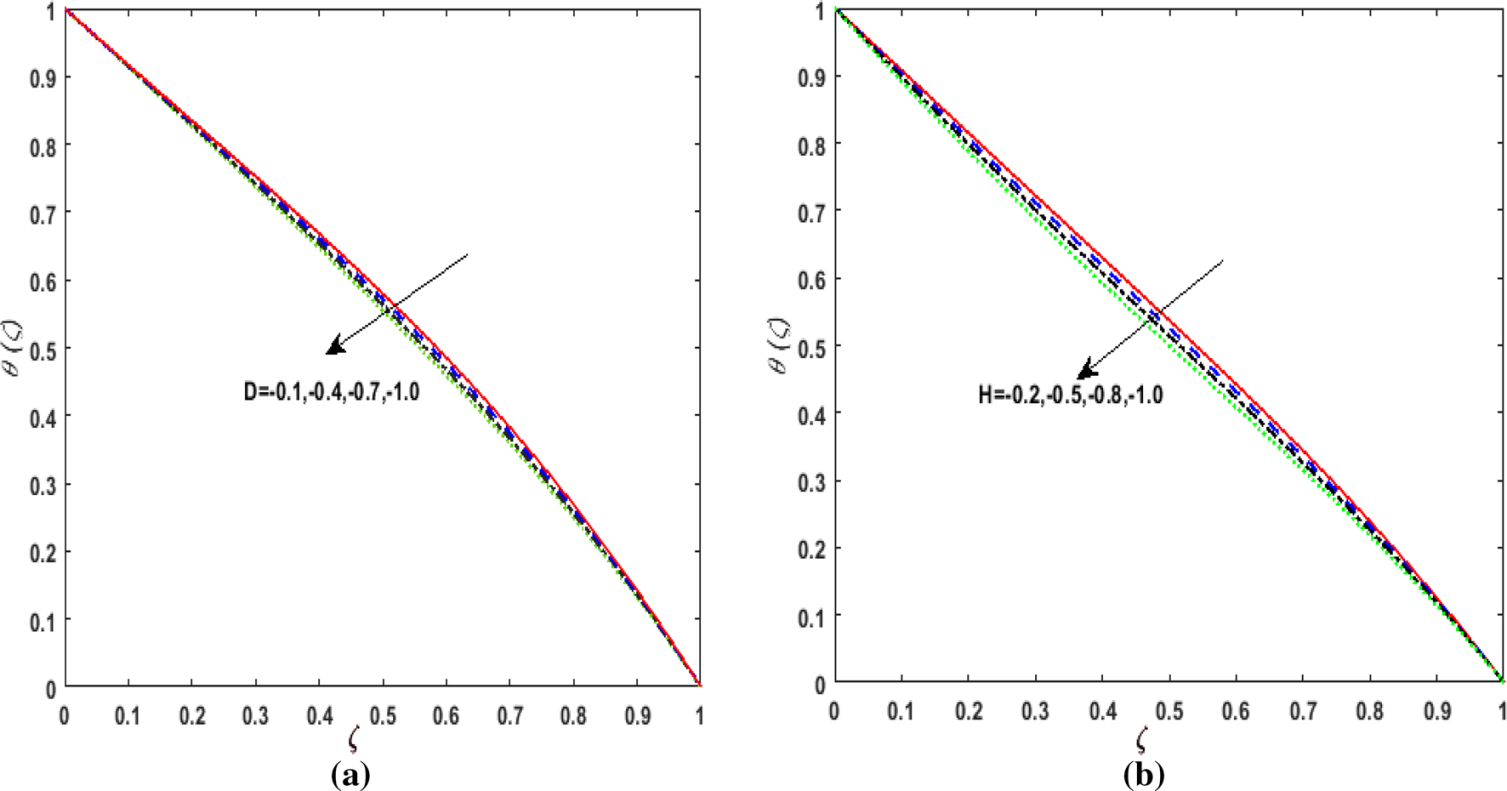
(**a**) Effect of temperature dependent sink parameter $D < 0$ on temperature $\theta \left( \zeta \right)$. (**b**) Effect of space dependent sink parameter $H < 0$ on temperature $\theta \left( \zeta \right)$.

The outcome of the thermophoresis parameter $N_{t}$ on the temperature field $\theta \left( \zeta \right)$ is depicted in Fig. 7. For growing values of $N_{t}$ nanoparticles move from hot to cold fluid. It is noticed that on enhancing $N_{t}$, thermophoretic force is strengthened. Hence, $\theta \left( \zeta \right)$ augments. The impression of $N_{b}$ on $\theta \left( \zeta \right)$ is portrayed in Fig. 8. It is noticed that rising values of $N_{b}$ results in amplified heat generation owing to the collision of nanoparticles. Hence, $\theta \left( \zeta \right)$ upsurges. To illustrate the behavior of the radiation parameter $Rd$ on $\theta \left( \zeta \right)$ Fig. 9 is drawn. Insertion of $Rd$ in temperature field boosts the random movement of nanoparticles. Therefore, more heat is generated as a result of the continuous collision. Hence, an upsurge is noticed in $\theta \left( \zeta \right)$. Figure 10

elucidates the effect of the thermal conductivity parameter $d$ on $\theta \left( \zeta \right)$. Larger values of $d$ enhances the heat function. Therefore, augmentation in $\theta \left( \zeta \right)$ is perceived. The performance of variable heat source and variable heat sink on $\theta \left( \zeta \right)$ is addressed in Figs. 11a, b and 12a, b. It is noticed that growing values of variable heat source parameter $\left( {D > 0,H > 0} \right)$ corresponds to additional heat generation. Hence, $\theta \left( \zeta \right)$ augments. However, on varying the variable sink parameter $\left( {D < 0,H < 0} \right)$ a deteriorating nature is displayed by $\theta \left( \zeta \right)$ due to internal heat absorption.

**Figure 13:**
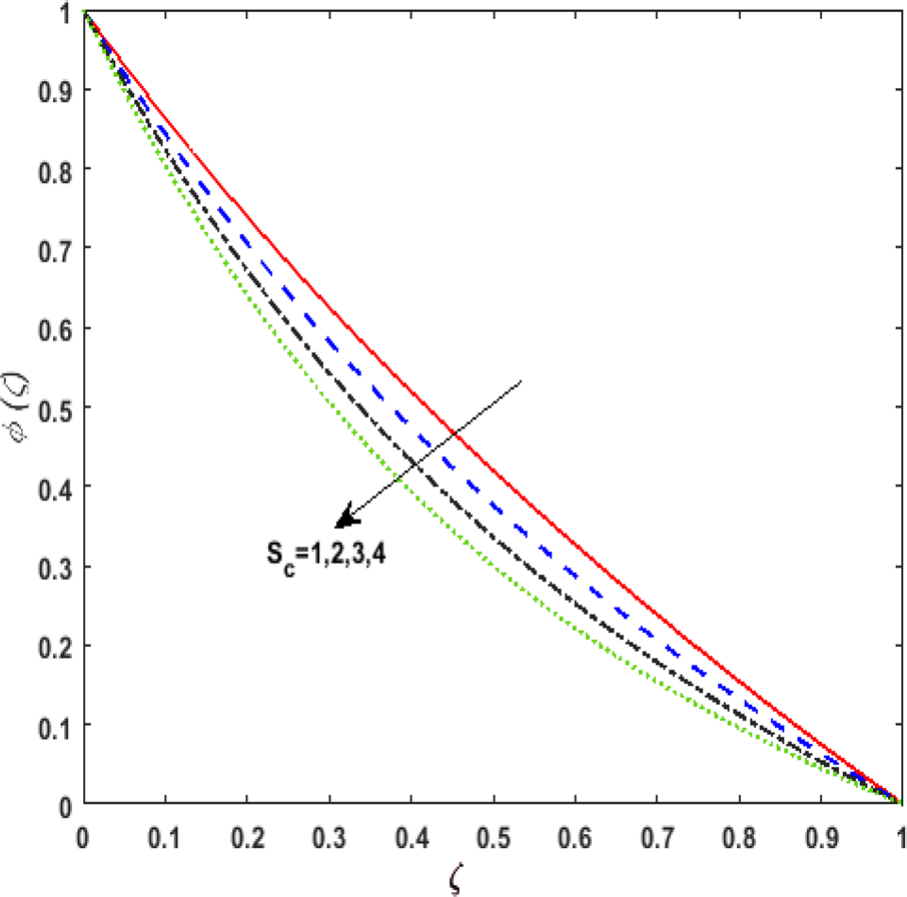
Effect of Schmidt number $S_{c}$ on concentration $\phi \left( \zeta \right)$.

**Figure 14:**
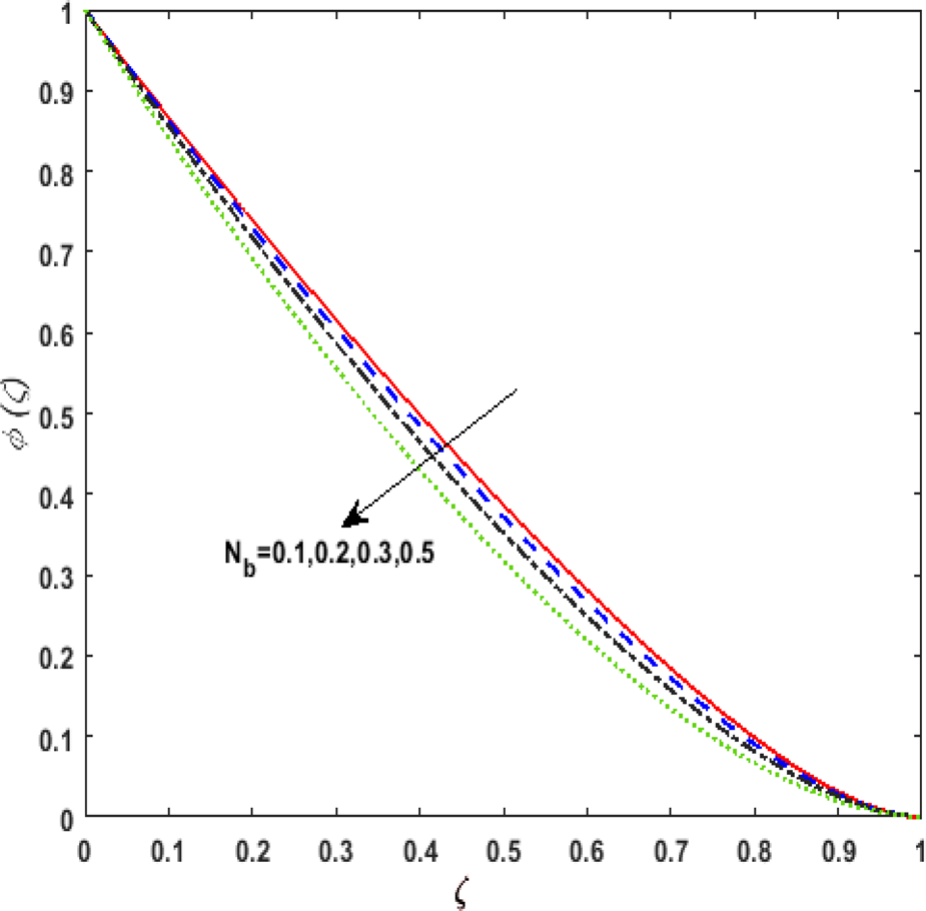
Effect of Brownian diffusion parameter $N_{b}$ on concentration $\phi \left( \zeta \right)$.

**Figure 15:**
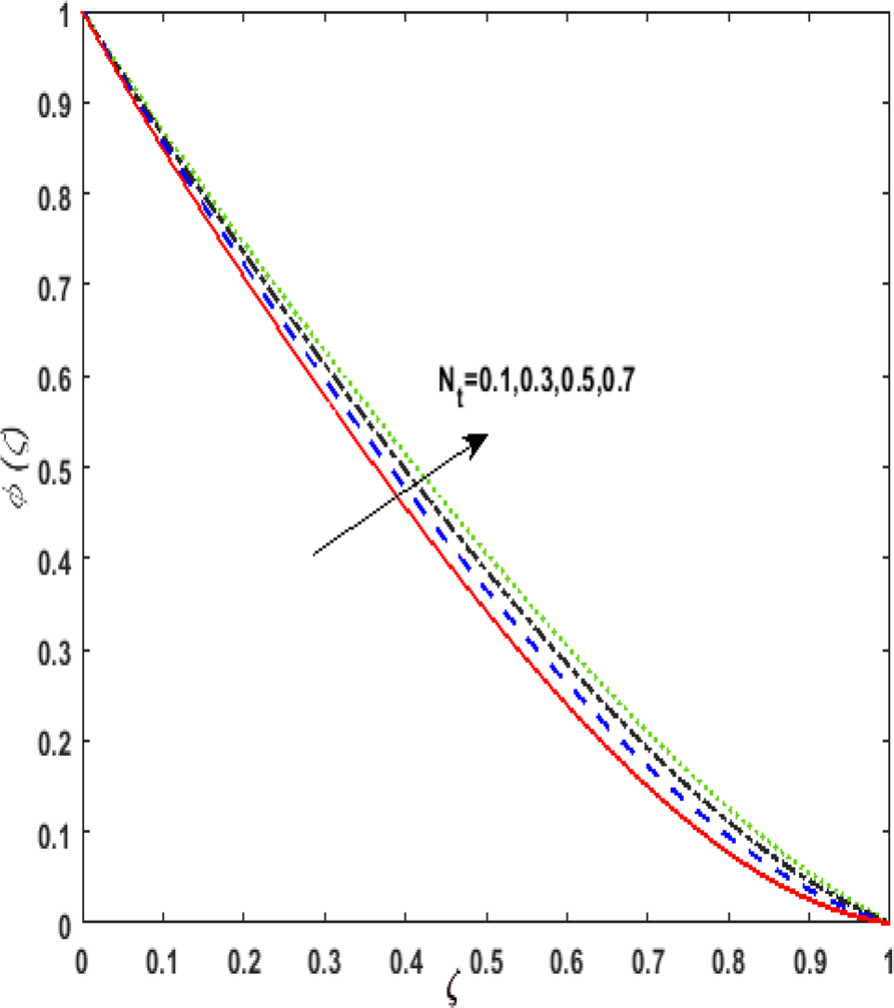
Effect of thermophoresis parameter $N_{t}$ on concentration $\phi \left( \zeta \right)$.

**Figure 16:**
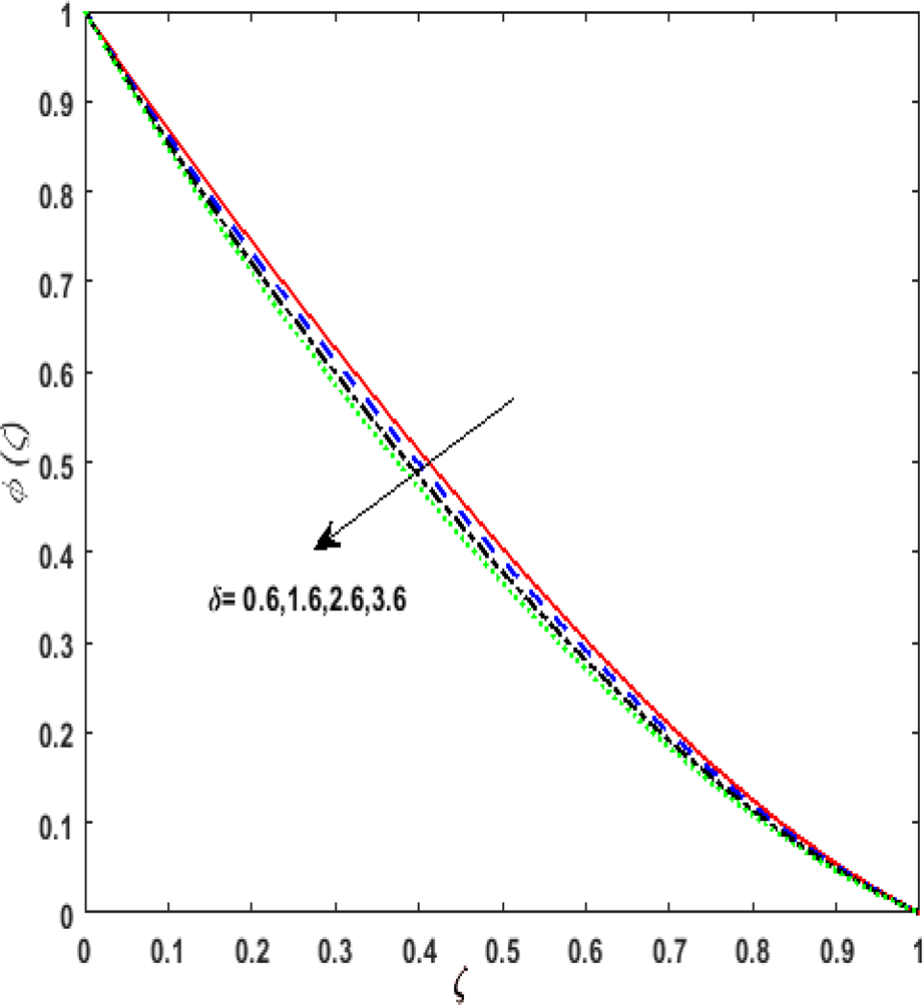
Effect of chemical reaction parameter $\delta$ on concentration $\phi \left( \zeta \right)$.

**Figure 17:**
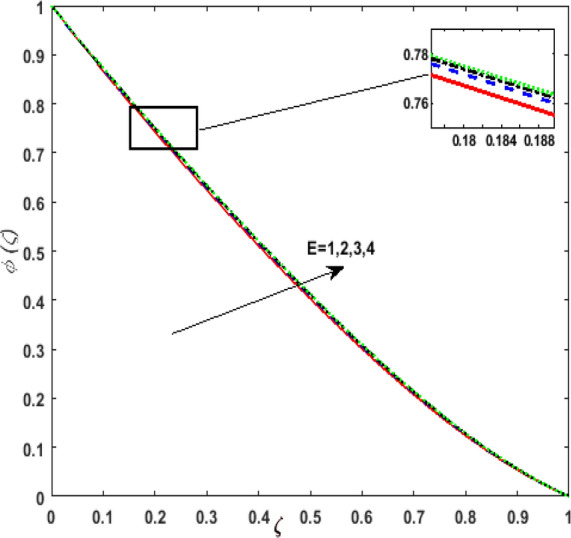
Effect of activation energy $E$ on concentration $\phi \left( \zeta \right)$.

**Figure 18:**
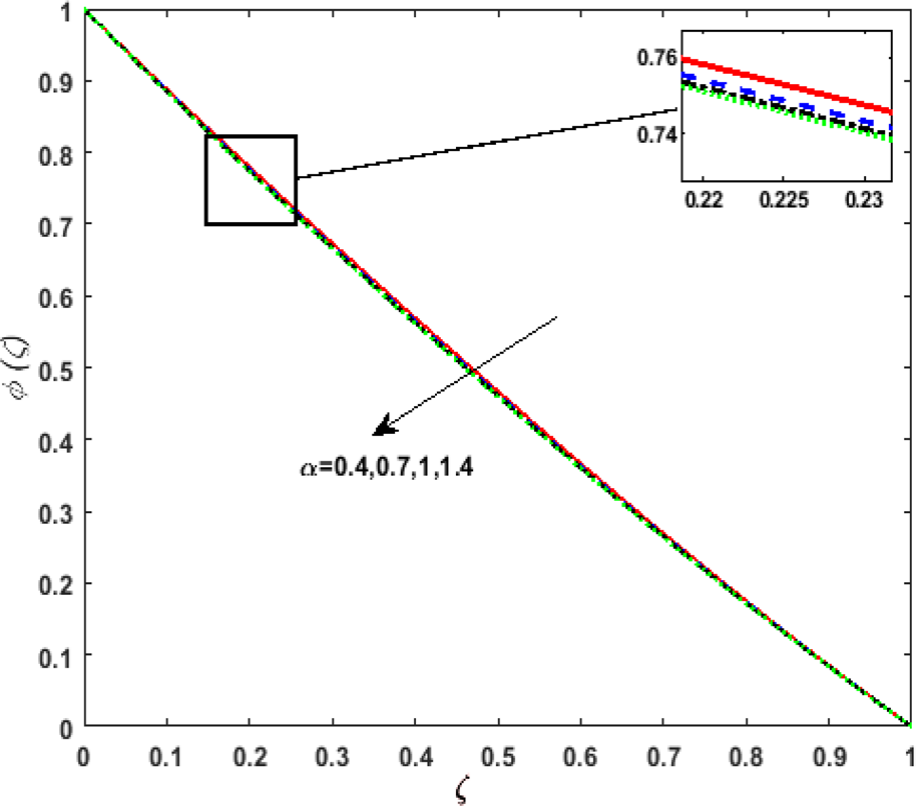
Effect of temperature difference parameter $\alpha$ on concentration $\phi \left( \zeta \right)$.

Figure 13 inspects the upshot of the Schmidt number $S_{c}$ on the concentration field $\phi (\zeta )$. It is noticed that by enhancing $S_{c}$ concentration profile decays due to the reduction of mass diffusion. The impact of the Brownian motion $N_{b}$ and thermophoresis parameter $N_{t}$ on $\phi (\zeta )$ is demarcated in Figs. 14 and 15. An opposing drift is perceived for $N_{b}$ and $N_{t}$ versus $\phi (\zeta ).$ Large values of $N_{t}$ fortifies the movement of fluid particles and thus $\phi (\zeta )$ upsurges. On escalating $N_{b}$, random movement augments among the fluid particles. Thus, amplifying $N_{b}$ fluid concentration decays. Figure 16 is drawn to elucidate the upshot of the dimensionless chemical reaction parameter $\delta$ on $\phi \left( \zeta \right)$. On upsurging $\delta ,$ chemical molecular diffusivity reduces owing to its consumption in the reaction. Hence, the concentration of fluid represses. The impression of mounting values of activation energy $E$ is deliberated in Fig. 17. It is noticed that escalating values of $E$ lead to a decrease in the Arrhenius function. Consequently, the generative chemical reaction decelerates. Thus, on escalating $E$, the fluid concentration upsurges. The outcome of the temperature difference parameter $\alpha$ on $\phi (\zeta )$ is portrayed in Fig. 18. As the temperature difference (at the surface and far away from the surface) increases a weaker concentration profile is witnessed.

The comportment of $Ha,\alpha_{1} {\text{ and }}K$ on shear stress at the lower and upper walls is examined in Table 2. It is perceived that on escalating $Ha$ shear stress increases at both walls. It also depicts that $f^{\prime\prime}(0),j^{\prime}(0)$ declines on mounting the values of $K,$ however, shear stress at the upper wall augments. On amplifying the rotation parameter shear stress at the upper wall escalates, whereas, a reverse impact is noted for $f^{\prime\prime}(0).$ The outcome of tabulated values of dimensionless parameters $\Pr ,Rd{\text{ and }}d$ on the heat transfer rate is depicted in Table 3. It is noticed that on escalating $\Pr ,Rd$ and $d$ heat flux augments. The outcome of numerous values of $S_{c} ,\delta ,E,N_{t}$ and $N_{b}$ on mass transfer rate is presented in Table 4. It is found that for growing values of $S_{c}$ and $\delta$ mass flux $\phi^{\prime}\left( 0 \right)$ augments at the lower wall. However, $\phi^{\prime}\left( 0 \right)$ decays for larger values of $E,N_{t} {\text{ and }}N_{b} .$ The mass transfer rate deteriorates at the upper wall for rising values of $S_{c}$ and $\delta ,$ though a reverse impact is witnessed for $E,N_{t} {\text{ and }}N_{b} .$ A comparative analysis of the present investigation is exhibited in Table 5 with Mohyud-Din et al.^48^. A good association between the results is seen.

**Table 2:** Computational values of friction drag coefficient at both walls against the different estimation of $Ha,\alpha_{1}$ and $K.$

$Ha$	$\alpha_{1}$	$K$	$f^{\prime\prime}\left( 0 \right)$	$j^{\prime}\left( 0 \right)$	$f^{\prime\prime}\left( 1 \right)$	$j^{\prime}\left( 1 \right)$
0.5	1	0.1	− 4.7151344	0.11530048	2.4815814	0.43738038
0.7			− 4.6872093	0.11826185	2.4881993	0.47997321
0.9			− 4.6508455	0.12031814	2.4976009	0.53976862
0.4	2	0.5	− 4.7877698	0.17561651	2.4942998	0.86176729
	4		− 4.9960522	0.26755264	2.5479799	1.4171942
	6		− 5.3056205	0.32703933	2.6358754	1.9474997
0.4	1	0.2	− 5.4333939	− 0.0222407	2.9982866	0.56661592
		0.4	− 6.9318571	− 0.2464316	3.9788672	0.82901674
		0.6	− 8.5521571	− 0.4198097	4.8832454	1.0604047

**Table 3:** Numeric values of the rate of heat transfer for growing values of $\Pr , \, Rd{\text{ and }}d$ at both walls.

$\Pr$	$Rd$	$d$	$- \left( {1 + \frac{Rd}{{1 + d\theta }}} \right)\theta ^{\prime}(0)$	$- \left( {1 + \frac{Rd}{{1 + d\theta }}} \right)\theta ^{\prime}(1)$
2	0.6	0.2	1.9592227	2.2686563
5			2.0121106	2.4056394
8			2.0652498	2.5491764
4	0.3	0.2	1.5926345	1.9745974
	0.5		1.8606061	2.2301873
	0.7		2.1282259	2.4889397
4	0.6	0.3	2.0691304	2.5778329
		0.5	2.2148867	3.0386267
		0.7	2.3568561	3.5308064

**Table 4:** Computational values of the rate of mass transfer for different estimations of $S_{c} ,N_{t} {, }\delta ,N_{b} {\text{ and E}}$ at both walls.

$S_{c}$	$N_{t}$	$\delta$	$N_{b}$	$E$	$- \phi ^{\prime}\left( 0 \right)$	$- \phi ^{\prime}\left( 1 \right)$
0.8	0.1	0.5	0.1	1	1.1571638	0.7671697
1.2					1.2458813	0.7330032
1.6					1.3360292	0.6998442
1.2	0.2				1.7903831	2.6215607
	0.3				1.6012781	2.9007375
	0.4				1.4268773	3.1963544
	0.2	0.7			1.1335845	0.7771404
		1			1.1636468	0.7660830
		1.3			1.1933839	0.7552334
			0.1		1.9944505	2.3592592
			0.3		1.5915746	2.8813571
			0.5		1.2517822	3.4622110
				2	1.0870076	0.7950733
				3	1.0742090	0.7998221
				4	1.0680021	0.8019768

**Table 5:** Comparison of temperature and concentration profile of present analysis with Mohyud-Din et al.^48^.

$\zeta$	Mohyud-Din et al.^48^$\theta \left( \zeta \right)$		Present	Mohyud-Din et al.^48^$\phi \left( \zeta \right)$		Present
	HAM	RK-4 method	bvp4c	HAM	RK-4 method	bvp4c
0	1	1	1	1	1	1
0.2	0.8679341	0.8679341	0.8679341	0.7270671	0.7270671	0.7270671
0.4	0.7082264	0.7082264	0.7082262	0.4847483	0.4847483	0.4847482
0.6	0.5157237	0.5157237	0.5157234	0.2782638	0.2782638	0.2782638
0.8	0.2829505	0.2829505	0.2829503	0.1137888	0.1137888	0.1137889
1	0	0	0	0	0	0

## Concluding remarks

Numerical solution for nanoliquid flow confined between two parallel infinite plates has been examined. The flow is analyzed with the combined impact of variable thermal conductivity, thermal radiation, and irregular heat source/sink. Mass transfer rate is incorporated with the impression of a chemical reaction and activation energy. The mathematical model is deciphered through MATLAB software bvp4c. The outcome of numerous parameters of the present investigation are:

- On escalating $K$, the velocity profiles diminish.
- For growing values of $N_{b} ,N_{t} ,d{\text{ and }}Rd$ an increasing behavior is depicted by the temperature field.
- The concentration field exhibits a reverse trend for $N_{b} {\text{ and }}N_{t}$.
- For larger values of $S_{c} {\text{ and }}\delta$ the concentration field declines.
- On amplifying the rotation parameter and suction parameter shear stress at the upper wall escalates.
- Heat transfer increases at the lower wall on amplifying $\Pr ,Rd$ and $d.$
- For higher values of $S_{c}$ and $\delta$ the mass transfer rate deteriorates at the upper wall, however, a reverse impact is witnessed on augmenting $E,N_{t}$ and $N_{b} .$

The subject manuscript may be extended to Hall current and Ion slip impacts amalgamated with any non-Newtonian fluid. The non-Newtonian fluids possess vast applications in the fluid arena. Furthermore, simple thermal radiation may be replaced with nonlinear thermal radiation and an effect of gyrotactic microorganisms may also be introduced.

## List of symbols

$c$: Stretching of the lower wall of the channel
$B_{0}$: Magnetic field strength
$$C$$: Fluid concentration
$C_{w}$: Lower plate concentration
$C_{l}$: Upper plate concentration
$$c_{p}$$: Specific heat
$D$: Temperature-dependent source/sink parameter
$D_{B}$: Brownian diffusion coefficient
$D_{T}$: Thermophoretic diffusion coefficient
$d$: Variable thermal conductivity parameter
$$E_{a}$$: Activation energy
$E = \frac{{E_{a} }}{\kappa T}$: Dimensionless activation energy
$H$: Space dependent source/sink parameter
$$Ha = \frac{{\sigma_{1} B_{0}^{2} h^{2} }}{\rho \nu }$$: Magnetic parameter
$k(T)$: Temperature-dependent thermal conductivity
$\overline{k}$: Mean absorption coefficient
$$K = \frac{{\nu_{0} }}{ch}$$: Suction parameter
$$m$$: Hall parameter
$n$: Fitted rate constant
$N_{b} = \frac{{\tau D_{b} \left( {C_{w} - C_{l} } \right)}}{\nu }$: Brownian motion parameter
$$N_{t} = \frac{{\tau D_{T} \left( {T_{h} - T_{l} } \right)}}{{T_{l} \nu }}$$: Thermophoresis parameter
$$\Pr = \frac{{\mu c_{p} }}{{k_{0} }}$$: Prandtl number
$$Q_{w}$$: Heat flux
$q_{r}$: Radiative heat flux
$$R_{e} = \frac{{ch^{2} }}{\nu }$$: Reynold number
${\text{Re}}_{h} = \frac{{u_{w} h}}{\nu }$: Local Reynold number based on channel height
$$Rd = \frac{{4\overline{\sigma }T_{l}^{3} }}{{\overline{k}k_{0} }}$$: Radiation parameter
$$S_{c} = \frac{\nu }{{D_{B} }}$$: Schmidt number
$$T$$: Fluid temperature
$T_{w}$: Upper plate temperature
$T_{l}$: Lower plate temperature
$u,v,w$: Component of velocity
$$x,y,z$$: Coordinate axes

## Greek symbols

$$\rho$$: Density
$$\nu$$: Kinematic viscosity
$$\sigma_{1}$$: Electrical conductivity
$$\Omega_{1}$$: Angular velocity
$\overline{\sigma }$: Stefan Boltzmann coefficient
$\delta = \frac{{k_{r}^{2} h^{2} }}{\nu }$: Chemical reaction parameter
$\alpha = \frac{{T_{w} - T_{l} }}{{T_{l} }}$: Temperature difference parameter
$$\alpha_{1} = \frac{{\Omega_{1} h^{2} }}{\nu }$$: Rotation parameter
$$\zeta$$: Similarity variable
